# Necessity and challenges for the post-pandemic Hangzhou Asian Games: An interdisciplinary data science assessment

**DOI:** 10.3389/fpsyg.2022.1047990

**Published:** 2022-12-27

**Authors:** Jianwei Guo, Xiangning Zhang, Dandan Cui

**Affiliations:** ^1^Institute of Artificial Intelligence in Sports, Capital University of Physical Education and Sports, Beijing, China; ^2^Beijing Institute for International Olympic Studies, Beijing, China

**Keywords:** Asian Games, COVID-19, elite sport, Olympic Games, public health

## Abstract

**Background:**

The postponement of the Hangzhou Asian Games has reignited controversy over whether it is necessary and safe to hold. This study aimed to assess its necessity for Asian elite sport and the challenges brought by the COVID-19 pandemic through joint data science research on elite sports and public health Internet big data.

**Methods:**

For necessity, we used seven pre-pandemic Asian Games to investigate its long-term internal balance and six pre-pandemic Olympic Games to examine its contribution to the external competitiveness of Asian sport powers through bivariate Pearson correlation analyses between sport variables and holding year. For challenges, we used Johns Hopkins COVID-19 data and Tokyo 2020 Olympic data to quantify the past impact of the pandemic on elite sport by another correlation analysis between pandemic variables and the change in the weighted score of medal share (CWSMS), built a transferable linear regression model, transferred the model to Jakarta 2018 Asian Games data, and eventually forecasted the possible impact of the pandemic on the results of the Hangzhou Asian Games.

**Results:**

The proportion of gold medal countries in the Asian Games showed a long-term upward trend (Pearson *r*_(7)_ = 0.849, *p* < 0.05), and the share of medals won by Asian countries showed a significant increasing process (Pearson *r*_(6)_ = 0.901, *p* < 0.05). The cumulative number of COVID-19 deaths (CND) was most significantly correlated to CWSMS (Pearson *r*_(100)_ = −0.455, *p* < 0.001). The total Olympic model output of Asian countries was 0.0115 in Tokyo 2020 and is predicted to be 0.0093 now. The prediction of CWSMS in Hangzhou was 0.0013 for China, 0.0006 for Japan, and 0.0008 for South Korea.

**Conclusion:**

We documented that Asian Games played a significant role in the long-term balanced internal structure and the increasing global competitiveness of Asian elite sport. We proved that the COVID-19 pandemic has significantly affected the Olympic performance of countries worldwide, while the competitive performance at the Hangzhou Games would be less affected than the world average level. This study also highlights the importance of interdisciplinary data science research on large-scale sports events and public health.

## 1. Introduction

On 6 May 2022, the Olympic Council of Asia (OCA) officially announced the postponement of the Hangzhou Asian Games due to the COVID-19 pandemic (Olympic Council of Asia, 2022). This is not the first time a major sport event has been postponed in the post-pandemic era. Since breaking out in early 2020, the global COVID-19 pandemic has affected global elite sport, so much so (Kemp et al., 2021; Hayes, 2022; Washif et al., 2022) that the Tokyo 2020 Olympics and Paralympics were postponed to 2021 for the first time in Olympic history (Olympic Games Tokyo Committee, 2021). Although both the Tokyo Olympics and the Beijing Winter Olympics were eventually held safely (International Olympic Committee, 2021a, 2022a; Akashi et al., 2022; Liu et al., 2022), public health risks caused by the new Omicron variant (Menni et al., 2022; Tanaka et al., 2022; Tian et al., 2022) and the attendance of spectators will undoubtedly bring new challenges to the Hangzhou Asian Games (Dergaa et al., 2022). As a result, public health and elite sport are more closely linked than ever (Kemp et al., 2021; International Olympic Committee, 2021b, 2022b), which has motivated many assessments on the impact of the pandemic on the Olympics. However, these assessments are either only quantitative from the perspective of elite sport (Csulak et al., 2021; Schipman et al., 2022) or only from the perspective of public health (Zhu et al., 2021; Akashi et al., 2022; Hirata et al., 2022), which cannot assess competitive performance with the dynamics of the pandemic. However, for a successful event, the excitement of the contests and the safety of its participants need to be achieved at the same time. To assess the possible challenges in Hangzhou Asian Games, interdisciplinary data science research that simultaneously quantifies the factors in elite sport and public health is required.

Not only the postponement but also the cancellation of large-scale sport events in the context of a global pandemic has been suggested (Borpujari, 2021; Lancet, 2021). In the case of the Asian Games, the controversy over its necessity began even earlier (Choi et al., 2015). Problems such as excessive scale, fixed mode, and a lack of marketing methods have labeled the Asian Games as “marginalized” (Choi et al., 2015). At the same time, China, Japan, and South Korea dominate the top three in the Asian Games; Hong proposed in his book that East Asian countries have formed a certain degree of “monopoly” in the Asian Games (Hong and He, 2020). Recently, Horne and Takahashi (2022) pointed out that the Asian Games has begun to be dubbed “the East Asian Games”. Before taking the risk, we need to evaluate whether it is necessary to hold the Games. But so far, most of the quantitative research around the Asian Games focuses on a certain event or a specific sport in a specific country (Lhee et al., 2021; Nanda et al., 2021). To assess the necessity of the Asian Games for the development of Asian elite sport, multi-level data science evidence around the overall long-term role of the Asian Games in Asian elite sport is required.

In fact, data science research on large-scale sport events has a long history: the first Olympic data analysis paper in *Science* (Lietzke, 1954) was published in 1954. In recent years, the rise of big data has led to the emergence of a new trend in Olympic data science research: multi-source and interdisciplinary. The joint analyses of Olympic data and social-economic data (Bernard and Busse, 2004; Forrest et al., 2010; Scelles et al., 2020), socio-demographic data (Smith et al., 2015; Guo et al., 2022), and even Google Trends data (Bauman et al., 2021) have provided quantitative bases for policy-making around elite sport, public economics, and public health throughout the Olympic bidding, preparing, and legacy managing (World Health Organization, 2010; Lee and Kim, 2013; Preuss, 2022; Russo et al., 2022), and have eventually deepened the integration between sport science and other disciplines such as econometrics, psychology, and medicine.

In this article, we aimed to provide quantitative evidence to assess the Hangzhou Asian Games in terms of its necessity for Asian elite sport and the challenges of the COVID-19 pandemic. The first part involved a multi-level investigation into the long-term pre-pandemic internal structure of Asian elite sport based on the historical data of the Asian Games and its external competitiveness based on the historical data of the Olympic Games. The second part also has two aspects. First, we conducted interdisciplinary modeling based on Johns Hopkins COVID-19 data and Tokyo 2020 Olympic data to assess the short-term impact of the pandemic on Asian and worldwide elite sport in terms of competitive performance and to forecast the possible pandemic impact on the contest results of the Hangzhou Asian Games. We also compared the multi-level dynamic pandemic data at the end in terms of infection risk.

## 2. Methods

Our study did not use any experimental subjects or private data, but mainly utilized big data from the Internet, which means that the institutional review board (IRB) and informed consent statement (ICS) are not involved. However, the collection and analysis of Internet big data are carefully designed.

### 2.1. Pre-pandemic necessity assessment

#### 2.1.1. Data collection

##### 2.1.1.1. Data of the Asian Games

Although the Asian Games have a long history, the main participating countries of the Asian Games were not fixed until some Central Asian countries that seceded from the former Soviet Union participated in the Hiroshima Asian Games in 1994. In addition, the last Asian Games before the outbreak of the COVID-19 pandemic were the Jakarta 2018 Asian Games. Therefore, we chose the historical data of the 7 Asian Games from 1994 to 2018 in our pre-pandemic assessment. From the official website of the OCA, we extracted the number of participating countries (NPCs), the number of medal-winning countries (NMCs), and the number of gold-medal-winning countries (NGMCs) in each Asian Games. Furthermore, the data on the number of gold, silver, and bronze medals won by China, Japan, and South Korea in each Asian Games were also collected.

##### 2.1.1.2. Data of the Olympic Games

Corresponding to the time period of the aforementioned Asian Games, we adopted the historical data of the six Olympic Games from 1996 to 2016 in our pre-pandemic assessment. On the official website of the International Olympic Committee (IOC), we extracted the total number of medals and gold medals, together with the total number of gold medals and medals won by 45 Asian National Olympic Committees (NOC) at each Olympic Games. Besides, the number of gold, silver, and bronze medals won by China, Japan, and South Korea in each Olympic Games was also collected.

In this article, data collection was performed using Python version 3.7.1 and pandas version 0.23.4. A summary of the data used in our pre-pandemic assessment is shown in Table 1.

**Table 1:** A summary of the data used in our pre-pandemic assessment.

	**Olympic Games**	**Asian Games**
Time interval	1996–2016	1994–2018
Number of sessions	6	7
Number of participating teams^a^	[197, 207]	[42, 45]
Number of gold medals^a^	[271, 307]	[310, 477]
Number of medals^a^	[842, 973]	[1,004, 1,552]

^a^Data are presented as a range.

#### 2.1.2. Statistical analysis

##### 2.1.2.1. Internal structure assessment

First, we calculated the proportion of gold-medal-winning countries (PGMC) and the proportion of medal-winning countries (PMC) in each Asian Games, together with the share of gold medals and medals of China, Japan, and South Korea. These variables can quantify the relative distribution of medals in different countries by excluding the interference from changes in the total number of participating countries (TNPCs) and the total number of medals (TNMs) in the Asian Games. Then, we analyzed the bivariate Pearson correlation between the aforementioned Asian Games variables and the year the Games were held by which the long-term development process and trends within Asia elite sport were quantified.

##### 2.1.2.2. External competitiveness assessment

For this problem, we first calculated the share of gold medals won by Asian countries (SGMAC) and the share of medals won by Asian countries (SMAC), together with the share of gold medals and medals of China, Japan, and South Korea in each Olympic Games. These variables can quantify the relative performance of Asian countries relative to the rest of the world by excluding interference from changes in Olympic TNPC or TNM. Then, we analyzed the bivariate Pearson correlation between the aforementioned Olympic Games variables and the year the Games were held. In this way, the long-term development process and trends of Asia elite sport in the global sport landscape have been quantified.

In this article, statistical analysis was performed using SPSS version 26.0 (IBM, NY, USA).

### 2.2. Post-pandemic challenge assessment

#### 2.2.1. Data collection

##### 2.2.1.1. Data of the Olympic Games

Since the Tokyo Olympic Games are the only comprehensive global summer sports event held since the COVID-19 breakout, the changes in the competitive performance of various countries during the Tokyo Olympics might reflect the short-term impact of the pandemic on their global competitiveness. Therefore, we chose the data from the Rio 2016 Olympics and the Tokyo 2020 Olympics for our post-pandemic assessment. On the official website of the IOC, we extracted the medal data of 103 medal-winning countries, regions, or organizations in the Rio or Tokyo Olympic Games, including the numbers of gold, silver, and bronze medals won by each of them.

##### 2.2.1.2. Data of the Asian Games

As the Jakarta 2018 Asian Games are the last in the pre-pandemic period, our forecast for the possible short-term impact of the pandemic on the results of the Hangzhou Asian Games is relative to the Jakarta 2018 Asian Games. Therefore, we chose the data from the Jakarta 2018 Asian Games as a control in our post-pandemic forecasting. On the official website of the OCA, we extracted the number of gold, silver, and bronze medals won by each of the 36 medal-winning countries or regions in the Jakarta 2018 Asian Games.

##### 2.2.1.3. Data of the COVID-19 pandemic

We obtained the cumulative number of confirmed COVID-19 cases (CNCCs) and the cumulative number of COVID-19 deaths (CND) in 100 of the Olympic medal-winning countries from the Center for Systems Science and Engineering (CSSE) at Johns Hopkins University (JHU) (Dong et al., 2020). In addition, pandemic-related data for the Democratic People's Republic of Korea and Turkmenistan were obtained from the official website of the World Health Organization (WHO). Particularly, the time point for the Olympic impact assessment data was 16 July 2021 (7 days before the Opening Ceremony of the Tokyo 2020 Olympic Games), and the time point for the Asian impact forecasting data was 1 May 2022.

Subsequently, in the *World Population Prospects 2019* released by the United Nations Population Division Department of Economic and Social Affairs in August 2019, we collected the population (2020) data of 100 medal countries or regions in the two Olympic Games. In addition, the population of Serbia and Kosovo was obtained from the Eurostat official website. The delegation of independent Olympic athletes with one national entity is excluded from our analysis.

A summary of the data used in our post-pandemic assessment is shown in Table 2.

**Table 2:** A summary of the data used in our post-pandemic assessment.

	**Olympic Games**	**Asian Games**
Games	Tokyo 2020	Jakarta 2018
Number of participating teams	207	45
Number of gold-medal-winning countries	65	28
Number of gold medals	340	465
Number of medal-winning countries	93	36
Number of medals	1,001	1,552
COVID-19 pandemic data collection date	Jul 16, 2021	May 1, 2022

#### 2.2.2. Statistical analysis

##### 2.2.2.1. Past impact documenting

To quantify the correlation between the change in the Olympic medal table from Rio to Tokyo and the COVID-19 pandemic, we need to define a variable to quantify the former first. Here, we introduced the weighted score of medal share (WSMS), which can quantify the relative performance of each country by always aligning with its medal table ranking and excluding the interference from changes in TNPC or TNM. Then, using the amount of change in WSMS from Rio to Tokyo (CWSMS), we quantified the change in the global competitive performance of each NOC in the post-pandemic era. The variable design is based on the following three points.

1) **Share:** The total number of medals is different for each Olympic Games and Asian Games. Therefore, we use the share of medals instead of the number of medals as a variable to compare the different competitions in history.
2) **Weight**: The ranking in the medal table is a recognized standard to measure the relative competitive performance of a country at the Olympic Games and Asian Games. From the perspective of ranking, the value of gold, silver, and bronze medals is different. First, the ranking of the medal table is determined by the number of gold medals. Second, in the case of the same number of gold medals, it is determined by the number of silver medals and so on. Therefore, we have weighted the share of medals in a way that can ensure that the scores of different countries are consistent with the ranking order of the medal table.
**3) Change**: In the long run, the competitive performance of countries is relatively stable, and the impact of the pandemic is reflected in the short-term change between the competitive performance of the two Olympic Games before and after the pandemic. Therefore, our dependent variable in the model is CWSMS rather than the original value.

(1) $$\begin{array}{l} WSMS_{}=g+s*0.001+b*0.001*0.001 \end{array}$$

As Equation 1 shows, *g* represents the gold medal share won by each country or region in the current Olympic Games, *s* represents the silver medal share, and *b* represents the bronze medal share. Besides CNCC and CND, we also calculated the cumulative number of CNCCs per unit population (CNCCP) and the cumulative number of confirmed COVID-19 deaths per unit population (CNDP) in each NOC. Considering the home advantage of host countries (Balmer et al., 2003), Brazil and Japan were excluded from the statistics. With no national entity, the delegation of independent Olympic athletes was also excluded.

Then, we analyzed the bivariate Pearson correlation between the aforementioned COVID-19 pandemic variables (on 16 July 2021) and CWSMS. In this way, the short-term impact of the pandemic on global elite sport at the Tokyo 2020 Olympic time point was quantified.

##### 2.2.2.2. Future result forecasting

Furthermore, taking CWSMS as the dependent variable and the COVID-19 pandemic variable that showed the strongest correlation with it as the independent variable, we trained a regression model on the Tokyo 2020 Olympic data and the pandemic data on 16 July 2021. To achieve the goal of effectively forecasting the results of the Hangzhou Asian Games, the model needs to meet the following three constraints.

1) Since the absolute value of the pandemic data inevitably increases with time, the model validity must not be affected by the increase in the absolute value of the pandemic data.
2) As the NPCs *n* in the Olympic Games and the Asian Games are different, the model validity must not be affected by changes in the total NPCs.
3) The total number of WSMS in a competition is always equal to 1.001001, so the total output of CWSMS from the model should be 0.

For constraint (1), the original value was replaced by its share form as the independent variable *X*; for constraints (2) and (3), we designed the following prototype of the model as Equation 2 shows.


(2) $$\begin{array}{l} CWSMS=a*\left(X-\frac{1}{n}\right) \end{array}$$

Then, we transferred the Olympic model to the Asian Games by bringing the COVID-19 pandemic data at the Asian impact time point (1 May 2022) of 36 gold-medal-winning countries at the Jakarta Asian Games into it and eventually built the regression model of the possible impact of the pandemic on the relative performance of the gold-medal-winning countries at the Hangzhou Asian Games.

In addition, we compared the dynamic changes of the pandemic variables CNCC and CND between the two time points. Based on this, we discussed the validity of the model outputs on two scales, Asia as a whole, and China, Japan, and South Korea as specific countries. The infection risks that may be caused by the new Omicron variant and the attendance of spectators were also explored.

## 3. Results

### 3.1. Pre-pandemic assessment

**Figure 1:**
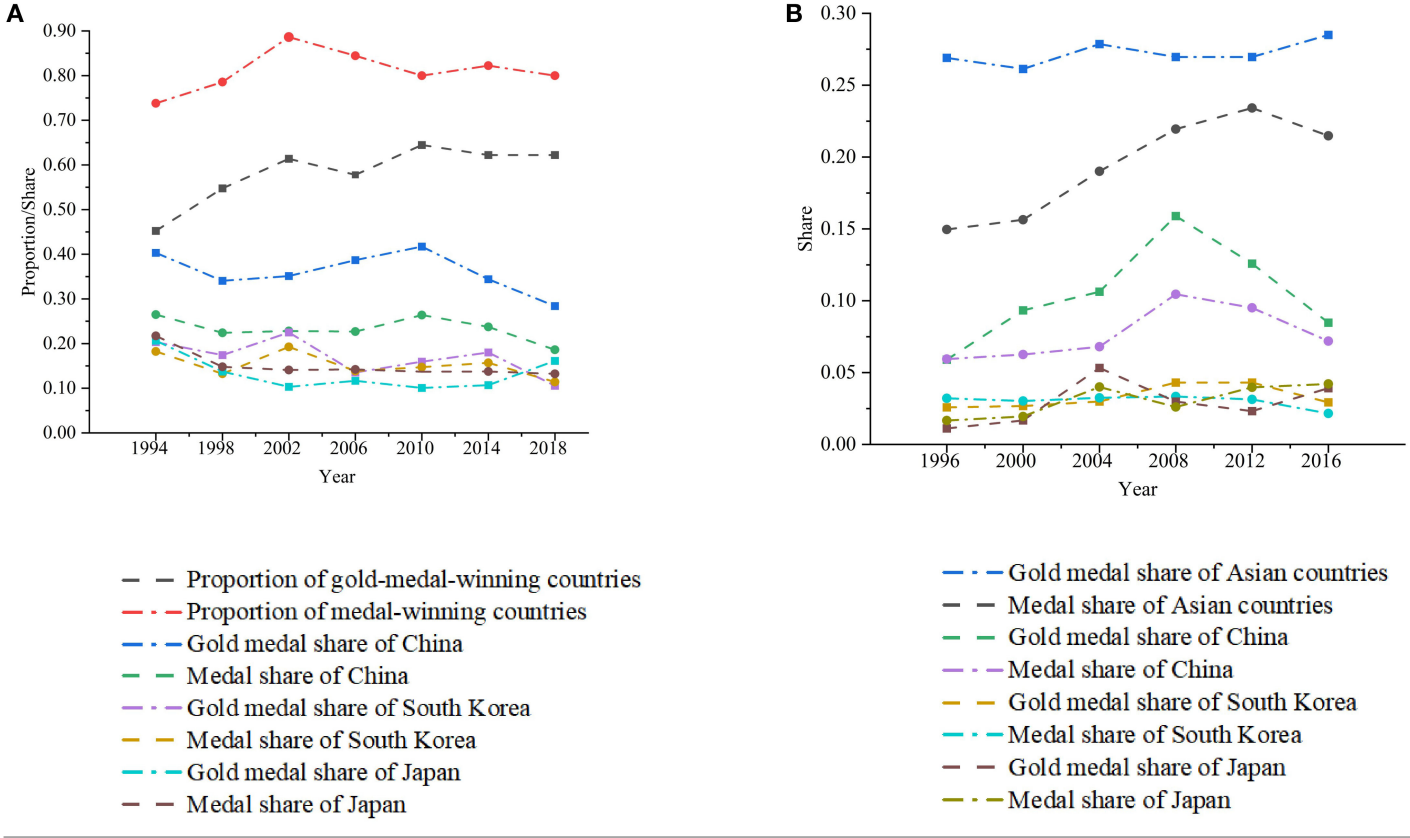
The results of the pre-pandemic assessment. **(A)** The internal trends in the Asian Games from 1994 to 2018 include the proportion of gold-medal-winning countries and the proportion of medal-winning countries, together with the share of gold medals and medals of China, Japan, and South Korea in each Asian Games. **(B)** The external competitiveness of Asian countries in the Olympic Games from 1996 to 2016 includes the share of gold medals won by Asian countries and the share of medals won by Asian countries, together with the share of gold medals and medals of China, Japan, and South Korea in each Olympic Games.

Within Asia, as shown in Figure 1A, at the continent level, in the 7 Asian Games from 1994 to 2018, PGMC showed a significant increasing process over time (Pearson *r*_(7)_ = 0.849, *P* < 0.05), while PMC had no significant upward trend. Conversely, at the particular country level, the medal and gold medal shares of China, Japan, and South Korea showed an overall downward trend but were not significant except for the medal share of Japan (Pearson *r*_(7)_ = −0.726, *P* < 0.1).

At the world level, as shown in Figure 1B, in the 6 Olympic Games from 1996 to 2016, SMAC showed a significant increasing process over time (Pearson *r*_(6)_ = 0.901, *P* < 0.05), and SGMAC also showed a significant upward trend (Pearson *r*_(6)_ = 0.742, *P* < 0.1). In contrast, at the particular country level, neither the share of gold medals nor the share of medals in China, Japan, and South Korea showed a significant correlation with time except for the medal share of Japan (Pearson *r*_(6)_ = 0.822, *P* < 0.05).

### 3.2. Post-pandemic assessment

For the past Olympic impact, at the world level, based on Olympic data and pandemic data at the first time point, the correlation analysis results showed that there were three COVID-19 pandemic variables significantly correlated with CWSMS, namely, CNCC (Pearson *r*_(100)_ = −0.400, *P* < 0.001), CND (Pearson *r*_(100)_ = −0.455, *P* < 0.001), and CNDP (Pearson *r*_(100)_ = −0.200, *P* < 0.05). CND was the most significant pandemic variable, so we adapted the share of CND (SCND) as the independent variable for regression analysis and trained an Olympic regression model as Equation 3 (a significant equation was found (*F*_(1, 99)_ = 24.006, *P* < 0.001) with adjusted *R*^2^ = 0.187) shows.


(3) $$\begin{array}{l} CWSMS_{Olympic}=-1.41e^{-1}SCND_{Olympic}+1.39e^{-3} \end{array}$$

**Figure 2:**
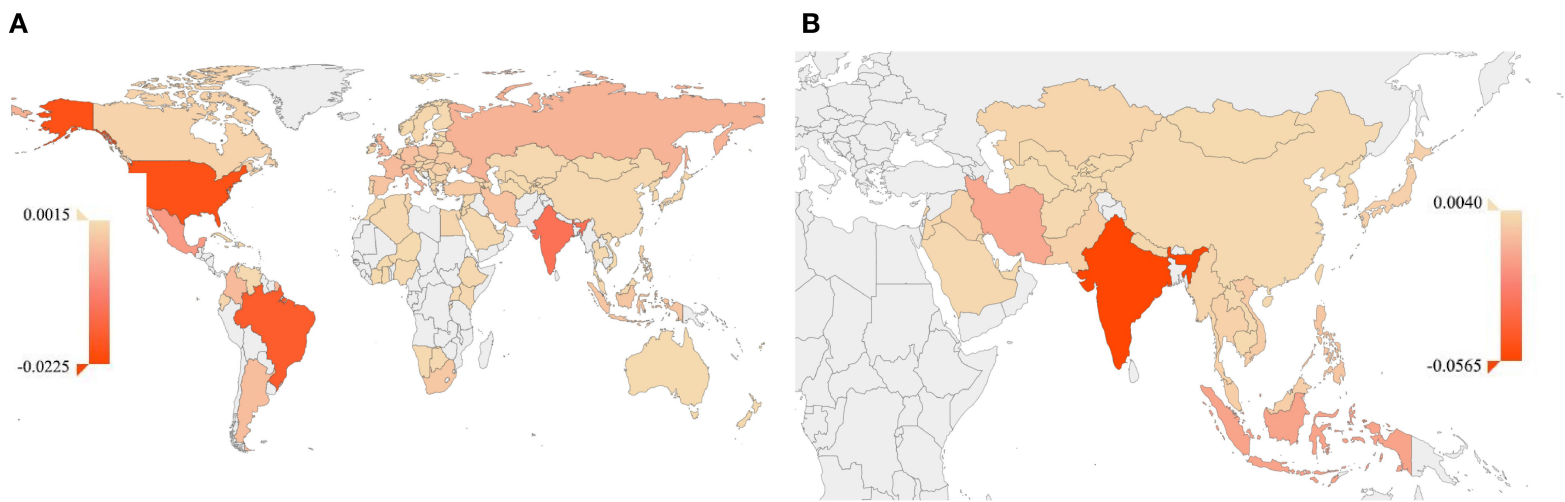
The results of the post-pandemic assessment. Yellow-red shading shows the CWSMS outputs of the model where darker red identifies countries in which the impact of the pandemic on WSMS is more severe. Light gray shading shows the regions that are not modeled. **(A)** The Olympic regression model outputs of CWSMS for 102 medal-winning countries from Rio 2016 to Tokyo 2020. **(B)** The Asian Games forecasting model outputs of CWSMS for 36 Jakarta gold-medal-winning countries in the Hangzhou Asian Games. WSMS, weighted score of medal share; CWSMS, change in the weighted score of medal share.

At the continent level, the sum of CWSMS model outputs for Asian countries was 0.0115. As shown in Figure 2A, at the particular country level, the Olympic model output of CWSMS for China was 0.0012, that of Japan was 0.0008, and that of South Korea was 0.0013. Moreover, according to the Olympic model in Equation 3, at the second time point (1 May 2022), the sum of CWSMS outputs for Asian countries was 0.0093, in general, and 0.0013 for China, 0.0006 for Japan, and 0.0008 for South Korea, in particular.

For the future Asian Games result, by bringing CND data at the second time point in 36 Jakarta gold-medal-winning countries into Equation 3, we built a regression model as Equation 4 shows.


(4) $$\begin{array}{l} CWSMS_{Asian}=-1.41e^{-1}SCND_{Asian}+3.93e^{-3} \end{array}$$

As shown in Figure 2B, the Asian Games model output of CWSMS for China was 0.0033, that of Japan was 0.0005, and that of South Korea was 0.0013.

Table 3 compares the absolute values and share forms of CNCC and CND for the world, Asia, China, Japan, and South Korea at the two time points.

**Table 3:** The absolute numbers and world shares of CNCC and CND for Asia, China, Japan, and South Korea at the two time points.

**Time point**	**Pandemic variable**	**World**	**Asia**		**China**		**Japan**		**South Korea**	
		**Number**	**Number**	**Share**	**Number**	**Share**	**Number**	**Share**	**Number**	**Share**
Jul 16, 2021	CNCC	190,042,343	58,627,144	3.08E-01	102,275	5.38E-04	834,303	4.39E-03	176,500	9.29E-04
	CND	4,076,664	839,769	2.06E-01	4,636	1.14E-03	15,029	3.69E-03	2,055	5.04E-04
May 1, 2022	CNCC	513,810,617	148,996,726	2.90E-01	1,002,919	1.95E-03	7,898,133	1.54E-02	17,295,733	3.37E-02
	CND	6,236,496	1,429,090	2.29E-01	5,092	8.16E-04	29,601	4.75E-03	22,958	3.68E-03

CNCC, cumulative number of confirmed COVID-19 cases; CND, cumulative number of COVID-19 deaths.

As CNCC increased sharply at the second time point in Table 3, we also built an alternative regression model that takes the share of CNCC (SCNCC) as the independent variable [a significant equation was found (*F*_(1, 99)_ = 18.556, *P* < 0.001) with adjusted *R*^2^ = 0.149]. The model is as follows.


(5) $$\begin{array}{l} CWSMS_{Olympic}=-1.13e^{-1}SCN{CC}_{Olympic}+1.11e^{-3} \end{array}$$

At the continent level, the sum of CWSMS alternative model outputs for Asian countries was 0.0008. At the particular country level, the alternative Olympic model output of CWSMS for China was 0.0010, that of Japan was 0.0006, and that of South Korea was 0.0010. Moreover, according to the alternative Olympic model in Equation 5, at the second time point (1 May 2022), the sum of CWSMS outputs for Asian countries was 0.0029, in general, and 0.0009 for China, −0.0007 for Japan, and −0.0030 for South Korea, in particular.

For the future Asian Games result, by bringing CNCC data at the second time point in 36 Jakarta gold-medal-winning countries into Equation 5, we built a regression model as Equation 6 shows.


(6) $$\begin{array}{l} CWSMS_{A\text{sian}}=-1.13{\text{e}}^{-1}SCNCC_{A\text{sian}}+3.13{\text{e}}^{-3} \end{array}$$

The alternative Asian Games model output of CWSMS for China was 0.0022, that of Japan was −0.0041, and that of South Korea was −0.0128.

## 4. Discussion

Regarding the role of the Asian Games in the overall balance and long-term development of Asian elite sport in the pre-pandemic period, we discussed it from both an intra-Asian perspective and a global perspective. Within Asia, the analysis of the Asian Games from 1994 to 2018 showed a significant increase in the proportion of medal countries and relative stability of the medal shares in China, Japan, and South Korea, which suggests that in the past two decades before the pandemic breakout, the rise of more sports powers has made the long-term development trend of the internal structure of Asian elite sport more balanced, which is inconsistent with the views of the “monopoly” of the 3 East Asian countries (Hong and He, 2020). In addition, the more intense competition also reflects that the Asian Games play a strong role in promoting the improvement of the competitive level of Asian countries to some extent. At the same time, this also overturns the so-called “monopoly” and “East Asian Games” (Horne and Takahashi, 2022).

Externally, in the analysis of Olympic data from 1996 to 2016, we found a significant increase in the share of medals won by 45 Asian countries and relatively stable medal shares in China, Japan, and South Korea in the six Olympic Games. This suggests that in the past two decades before the pandemic breakout, the overall competitiveness of Asian elite sport in the world sports arena has steadily improved. This further implies that the aforementioned emergence of more Asian Games medal-winning countries is not because of the weakening of the major Asian sport powers. On the contrary, at the particular country level, when China, Japan, and South Korea hold a stable position in the world elite sport arena, other Asian sports powers are gradually emerging, which in turn enhances the overall competitiveness of Asian elite sport in the world arena.

Moreover, linking the internal and external trends together, if we consider China, Japan, and South Korea as a frame of reference, the rise of other Asian sports powers is consistent with the overall competitiveness of Asian elite sport in the world arena. According to Figures 1A, B, the upward trend of the Asian Games is ahead of the upward trend of the Olympic Games. In other words, the prosperity of the Asian Games precedes the prosperity of Asian elite sport powers in the Olympic Games. That is to say, in the long-term development process before the pandemic, the balanced inward strength of Asian elite sport embodied by the Asian Games also indirectly enhanced the outward world competitiveness of Asian elite sport, which can hardly be named “marginalized” (Choi et al., 2015).

Here, a possible alternative explanation is that the pre-pandemic statistical analysis did not exclude the host country's advantage, which is also a possible reason for the declining trend in Japan's gold medal share during the selected time range. In fact, we intentionally included it because hosting large-scale comprehensive international events is also one of the effective measures for a country to improve its elite sport systematically and rapidly, for which China has set a good example (Zheng and Chen, 2016). Holding the Asian Games in different countries will promote the rapid rise of more Asian sports powers, for which Qatar has also set a good example (Abdul Razak and Muhamad, 2022). This is another important contribution of the Asian Games to the long-term and balanced development of Asian elite sport.

Regarding the impact of the COVID-19 pandemic on global elite sport, before the Tokyo 2020 Olympics, the number of confirmed cases and deaths in each country was significantly correlated with the change in its ranking in the Olympic medal table. This suggests that the pandemic has indeed affected the overall short-term development of elite sport in various countries around the world, which is consistent with the former discipline-specific conclusions (Schipman et al., 2022) and pre-Olympic reports (Kemp et al., 2021; Washif et al., 2022).

Therefore, the joint modeling of Olympic data and the pandemic data may forecast the impact of the COVID-19 pandemic on the performance of elite sport in various countries to some extent. According to our results, at the continent level, the overall impact on Asian participating countries is lower than the world average in terms of Tokyo Olympic performance, which also implies that Asian countries may present a relatively more exciting post-pandemic event in the upcoming future at the Hangzhou Asian Games. The reason may lie in the fact that Asian sports powers (Wang et al., 2021), especially China (Chen et al., 2021), have taken more effective COVID-19 countermeasures, and the fact that most Asian countries are less urbanized than European and American countries (González-Val and Sanz-Gracia, 2022). Particularly, China, Japan, and South Korea are less affected by the COVID-19 pandemic than the world and Asian average levels no matter from the output of the Olympic regression model at the Tokyo time point or the forecasting of the Asian Games regression model at the second time point. In other words, they may achieve better results in the 2022 Hangzhou Asian Games than in the 2018 Jakarta Asian Games.

The validity of the above predictions can be verified by two of our complementary analyses. On the one hand, bringing the pandemic data in May 2022 into Equation 3, the output of Asia, China, Japan, and South Korea is still positive, which suggests that our judgment based on the Tokyo data is still basically valid for now. On the other hand, the milder symptoms and higher infection rate of the Omicron variant relative to the Delta variant (Menni et al., 2022) resulted in a faster increase in confirmed cases and a slower increase in deaths across all scales in Table 3, which also implies that CNCC may replace CND as the primary factor affecting elite sport due to the pandemic. As the output of the alternative model shows, the CNCC-based predictions for the relative competitive performance of Asia as a whole and China, in particular, remain positive, but the output for Japan and South Korea turns negative. Although this does not affect our prediction of how exciting the Asian Games will be, there are more possibilities for the relative performance of countries in the upcoming Hangzhou Asian Games. If CND remains the primary factor, the pandemic will strengthen the relative performance of China, Japan, and South Korea in Hangzhou; otherwise, if CNCC becomes the leading factor, the relative performance of Japan and South Korea will be weakened due to the sharp increase of CNCC in Omicron wave. Furthermore, the slight increase in CND share in Asia in Table 3 may be due to the fact that Omicron's later arrival in Asia and its slower dispersion there have delayed the variant's impact when compared with Europe and North America (Elliott et al., 2022; Taylor, 2022), which are also leading continents of elite sport.

Besides the impact of the pandemic on competitive performance, the infection risk brought by the attendance of spectators is more complex. The higher infection rate does bring higher public health risks to spectators watching the Games (Dergaa et al., 2022), but it cannot be ignored that the vast majority of the staff and spectators of the Games will come from Asia, especially China. The shares of CNCC and CND in Table 3 on both time points are far lower than the share of the world's population in Asia and China according to the UN population data we collected (58.93%). This implies that even if the Games is open to spectators, the risk is lower than that of recent Games in other countries or continents.

Our study has two major limitations. First, the challenge caused by the attendance of spectators is not included in the prediction model. Nevertheless, our interdisciplinary modeling with the help of dynamic pandemic big data has partially made up for it. Furthermore, we will continue to follow up and investigate the Qatar World Cup (Dergaa et al., 2022), which may provide new evidence for our further research. Second, the impact of the pandemic during the Omicron wave may change (Menni et al., 2022). The comparative analysis of pandemic variables between Delta and Omicron using an alternative model has included more possibilities. Moreover, the current insufficiency is precisely the motivation for our continued interdisciplinary tracking of elite sport and public health big data in the future.

## 5. Conclusion

Our study shows that during the two decades before the pandemic, the internal structure of Asian elite sport showed a trend of more balanced medal distribution in the Asian Games. Consequently, at the world level, the overall competitiveness of Asian countries shows a significant trend of strengthening in the Olympic Games. The Asian Games play a significant role in the long-term development of Asian elite sport at multiple levels, and its holding is necessary.

About 2 years after the outbreak of the pandemic, the joint modeling on the Tokyo 2020 Olympic Games and the Johns Hopkins COVID-19 data shows that the impact of the pandemic on competitive performance does exist, but its impact on the Asian Games is lower than the world average level. Combined with the dynamic changes of the pandemic, we can forecast that the public health risk to the Hangzhou Asian Games is expected to be more controllable than other mega sport events elsewhere. Particularly, China, Japan, and South Korea, the three dominant sports powers in Asia, are still likely to perform better in Hangzhou than in the 2018 Jakarta Games. In contrast, given the well-documented impact of the pandemic on elite sport performance with high statistical significance but a small effect size, our advice to policymakers is to take different risk management among athletes and spectators while opening live audiences to ensure both excitement and safety, rather than opening them up entirely at once. We hope this can provide a reference for future large-scale sport events to be held in the prolonged post-pandemic era.

Another highlight of our study is the importance of deeper integration between public health and elite sport in the post-pandemic era. Moreover, an effective way to deepen their integration is through interdisciplinary data science research. Today, we can easily obtain data on large-scale sport events and real-time global pandemics through the Internet, but their joint analysis has also put forward new requirements for study design, data acquisition, and data integration. From the perspective of elite sport, data collection should not be limited to a single event or a single sport, but should be analyzed by combining multi-level data from multiple resources; from the perspective of public health, the pandemic data need to be dynamically quantified and modeled simultaneously with the sports data.

On 19 July, the OCA announced that the 19th Asian Games in Hangzhou will be held from 23 September to 8 October 2023. The Asian Games, postponed for 1 year, will bring us exciting sport and provide valuable data for both elite sport and public health for in-depth future assessment. We will continue to follow up on this issue in our future work.

## Data availability statement

Publicly available datasets were analyzed in this study. This data can be found here: https://coronavirus.jhu.edu/map.html; https://olympics.com/ioc/overview.

## Author contributions

JG contributed to data collection. DC and JG participated in the design of the study and contributed to data analysis. DC and XZ contributed to the interpretation of results. JG and DC contributed to the manuscript writing. All authors have read, approved the final version of the manuscript, and agreed with the order of presentation of the authors.

## References

[B1] Abdul Razak S. N. Muhamad T. A. (2022). Strategic sports planning in Malaysia and Qatar. Malaysian J. Sport Sci. Recreat. 18, 49–65. 10.24191/mjssr.v18i1.17639

[B2] Akashi H. Shimada S. Tamura T. Chinda E. Kokudo N. (2022). SARS-CoV-2 infections in close contacts of positive cases in the Olympic and Paralympic village at the 2021 Tokyo Olympic and Paralympic Games. JAMA. 327, 978–980. 10.1001/jama.2022.0818 35113161 PMC8814958

[B3] Balmer N. J. Nevill A. M. Williams A. M. (2003). Modelling home advantage in the summer Olympic Games. J. Sports Sci. 21, 469–478. 10.1080/0264041031000101890 12846534

[B4] Bauman A. E. Kamada M. Reis R. S. Troiano R. P. Ding D. Milton K.. (2021). An evidence-based assessment of the impact of the Olympic Games on population levels of physical activity. Lancet 398, 456–464. 10.1016/S0140-6736(21)01165-X 34302766

[B5] Bernard A. B. Busse M. R. (2004). Who wins the Olympic Games economic resources and medal totals. Rev. Econ. Stat. 86, 413–417. 10.1162/003465304774201824

[B6] Borpujari P. (2021). How the pandemic Olympics affected Japan. BMJ. 374:n2102. 10.1136/bmj.n2102 34452882

[B7] Chen H. Shi L. Zhang Y. Wang X. Sun G. A. (2021). Cross-country core strategy comparison in China, Japan, Singapore and South Korea during the early Covid-19 pandemic. Global. Health 17, 22. 10.1186/s12992-021-00672-w 33618688 PMC7897890

[B8] Choi C. Shin M. Kim C.-.G. (2015). Globalization, regionalism and reconciliation in South Korea's Asian games. Int. J. Hist. Sport 32, 1308–1320. 10.1080/09523367.2015.1031116

[B9] Csulak E. Petrov Á. Kováts T. Tokodi M. Lakatos B. Kovács A.. (2021). The impact of COVID-19 on the preparation for the Tokyo Olympics: a comprehensive performance assessment of top swimmers. Int. J. Environ. Res. Public Health 18, 9770. 10.3390/ijerph18189770 34574691 PMC8472124

[B10] Dergaa I. Saad H. B. Souissi A. Musa S. Abdulmalik M. A. Chamari K.. (2022). Olympic Games in COVID-19 times: lessons learned with special focus on the upcoming Fifa World Cup Qatar 2022. Br. J. Sports Med. 56, 654–656. 10.1136/bjsports-2021-105276 35232751

[B11] Dong E. Du H. Gardner L. (2020). An interactive web-based dashboard to track COVID-19 in real time. Lancet Infect. Dis. 20, 533–534. 10.1016/S1473-3099(20)30120-1 32087114 PMC7159018

[B12] Elliott P. Eales O. Steyn N. Tang D. Bodinier B. Wang H.. (2022). Twin peaks: the Omicron SARS-CoV-2 Ba.1 and Ba.2 epidemics in England. Science, 376:eabq4411. 10.1126/science.abq4411 35608440 PMC9161371

[B13] Forrest D. Sanz I. Tena J. D. (2010). Forecasting national team medal totals at the summer Olympic Games. Int. J. Forecast. 26, 576–588. 10.1016/j.ijforecast.2009.12.007

[B14] González-Val R. Sanz-Gracia F. (2022). Urbanization and COVID-19 incidence: a cross-country investigation. Paper Reg. Sci. 101, 399–415. 10.1111/pirs.12647

[B15] Guo C. Hu X. Xu C. Zheng X. (2022). Association between Olympic Games and children's growth: evidence from China. Br. J. Sports Med. 2021:104844. 10.1136/bjsports-2021-104844 35241433 PMC9484364

[B16] Hayes M. (2022). Social media and inspiring physical activity during COVID-19 and beyond. Manag. Sport Leis. 27, 14–21. 10.1080/23750472.2020.1794939

[B17] Hirata A. Kodera S. Diao Y. Rashed E. A. (2022). Did the Tokyo Olympic Games enhance the transmission of COVID-19? An interpretation with machine learning. Comput. Biol. Med. 146, 105548. 10.1016/j.compbiomed.2022.105548 35537221 PMC9040411

[B18] Hong F. He G. (2020). The Asian Games, Asian Sport and Asian Politics. The Routledge Handbook of Sport in Asia. London; New York, NY: Routledge, 493–504. 10.4324/9780429061202-52

[B19] Horne J. Takahashi Y. (2022). Mobile mega-event expertise in an “east asian era”. Sociol. Sport J. 36, 1–10. 10.1123/ssj.2020-0115

[B20] International Olympic Committee (2021a). New Data Shows No Covid-19 Spread between Tokyo 2020 Participants and Local Population. Available online at: https://olympics.com/ioc/news/new-data-shows-no-covid-19-spread-between-tokyo-2020-participants-and-local-population

[B21] International Olympic Committee (2021b). Tokyo 2020, A Global Health Effort That's Given Hope to the World. Available online at: https://olympics.com/ioc/news/tokyo-2020-a-global-health-effort-that-s-given-hope-to-the-world

[B22] International Olympic Committee (2022a). Ioc President's Speech – Beijing 2022 Closing Ceremony. Available online at: https://olympics.com/ioc/news/ioc-president-s-speech-beijing-2022-closing-ceremony

[B23] International Olympic Committee (2022b). Ioc and Who Reaffirm Collaboration to Promote Vaccine Equity and Healthy Lifestyles. Available online at: https://olympics.com/ioc/news/ioc-and-who-reaffirm-collaboration-to-promote-vaccine-equity-and-healthy-lifestyles

[B24] Kemp S. Cowie C. M. Gillett M. Higgins R. Hill J. Iqbal Z.. (2021). Sports medicine leaders working with government and public health to plan a ‘return-to-sport'during the COVID-19 pandemic: the Uk's collaborative five-stage model for elite sport. Br. J. Sports Med. 55, 4–5. 10.1136/bjsports-2020-102834 32661129

[B25] Lancet T. (2021). We Need a Global Conversation on the 2020 Olympic Games. Lancet. 397, 2225. 10.1016/S0140-6736(21)01293-9 34119048 PMC9752787

[B26] Lee Y. -H. Kim J. M. (2013). Olympic health legacy; essentials for lasting development of host city. J. Lifestyle Med. 3, 9–18. 26064832 PMC4390750

[B27] Lhee S. -H. Jain R. Sadasivam M. M. Kim S. Bae M.. (2021). Sports injury and illness incidence among South Korean elite athletes in the 2018 asian games: a single-physician prospective study of 782 athletes. BMJ Open Sport Exerc Med. 7:e000689. 10.1136/bmjsem-2019-000689 33614125 PMC7871279

[B28] Lietzke M. H. (1954). An analytical study of world and olympic racing records. Science. 119, 333–336. 10.1126/science.119.3089.333 17756808

[B29] Liu J. Lou J. Wang Y. Zhang J. (2022). Risk management strategies for the 2022 winter Olympic Games: the Beijing scheme. J. Sport. Health Sci. 2022, S2095–S2546. 10.1016/j.jshs.2022.02.006 35247620 PMC9532588

[B30] Menni C. Valdes A. M. Polidori L. Antonelli M. Penamakuri S. Nogal A.. (2022). Symptom prevalence, duration, and risk of hospital admission in individuals infected with SARS-CoV-2 during periods of omicron and delta variant dominance: a prospective observational study from the ZOE COVID study. Lancet 399, 1618–1624. 10.1016/S0140-6736(22)00327-0 35397851 PMC8989396

[B31] Nanda F. A. Novriansyah N. Nugroho M. D. Fajaruddin S. Utama M. B. R. Burhaein E.. (2021). Psychological skills of basketball athletes by perspektive gender: study Indonesian athletes in Asian Games Xviii. Sport Sci. 15.

[B32] Olympic Council of Asia (2022). Oca Press Release on Hangzhou Asian Games 2022 and Shantou Asian Youth Games 2021. Available online at: https://ocasia.org/news/2987-oca-press-release-on-hangzhou-asian-games-2022-and-shantou-asian-youth-games-2021.html

[B33] Olympic Games Tokyo Committee (2021). Review of Tokyo 2020. Available online at: https://www.tokyo2020.jp/ja/%E6%9D%B1%E4%BA%AC2020%E5%A4%A7%E4%BC%9A%E3%81%AE%E6%8C%AF%E3%82%8A%E8%BF%94%E3%82%8A%E3%81%AB%E3%81%A4%E3%81%84%E3%81%A6.pdf

[B34] Preuss H. (2022). Re-analysis, measurement and misperceptions of cost overruns at Olympic Games. Int. J. Sport Policy Politic. 14, 381–400. 10.1080/19406940.2022.2037685

[B35] Russo E. Figueira A. R. Mataruna-dos-Santos L. J. (2022). COVID-19, sustainability and Olympic Games: Which lessons can we learn from Tokyo 2020? *Sport Bus. Manag. Int. J*. 10.1108/SBM-09-2021-0109. [Epub ahead of print].

[B36] Scelles N. Andreff W. Bonnal L. Andreff M. Favard P. (2020). Forecasting national medal totals at the summer Olympic Games reconsidered. Soc. Sci. Q. 101, 697–711. 10.1111/ssqu.12782

[B37] Schipman J. Saulière G. Marc A. Hamri I. Rivallant Y. Difernand A.. (2022). The COVID-19 pandemic impact on the best performers in athletics and swimming among paralympic and non-disabled athletes. J. Sports Med. Phys. Fitness. 62, 1605–1614. 10.23736/S0022-4707.22.13365-7 35179330

[B38] Smith N. R. Lewis D. J. Fahy A. Eldridge S. Taylor S. J. Moore D. G.. (2015). Individual socio-demographic factors and perceptions of the environment as determinants of inequalities in adolescent physical and psychological health: the olympic regeneration in East London (Oriel) study. BMC Public Health 15, 1–18. 10.1186/s12889-015-1459-1 25884502 PMC4339478

[B39] Tanaka H. Ogata T. Shibata T. Nagai H. Takahashi Y. Kinoshita M.. (2022). Shorter incubation period among COVID-19 cases with the ba.1 omicron variant. Int. J. Environ. Res. Public Health 19, 6330. 10.3390/ijerph19106330 35627870 PMC9140418

[B40] Taylor L. (2022). Covid-19: omicron drives weekly record high in global infections. BMJ. 376:o66. 10.1136/bmj.o66 35017144

[B41] Tian D. Sun Y. Xu H. Ye Q. (2022). The emergence and epidemic characteristics of the highly mutated SARS-CoV-2 Omicron variant. J. Med. Virol. 94, 2376–2383. 10.1002/jmv.27643 35118687 PMC9015498

[B42] Wang X. Shi L. Zhang Y. Chen H. Sun G. (2021). Policy disparities in fighting COVID-19 among Japan, Italy, Singapore and China. Int. J. Equity Health 20, 33. 10.1186/s12939-020-01374-2 33441144 PMC7804586

[B43] Washif J. A. Farooq A. Krug I. Pyne D. B. Verhagen E. Taylor L.. (2022). Training during the COVID-19 lockdown: knowledge, beliefs, and practices of 12,526 athletes from 142 countries and six continents. Sports Med. 52, 933–948. 10.1007/s40279-021-01573-z 34687439 PMC8536915

[B44] World Health Organization (2010). The Health Legacy of the 2008 Beijing Olympic Games: Success and Recommendations.

[B45] Zheng J. Chen S. (2016). Exploring China's success at the Olympic Games: a competitive advantage approach. Eur. Sport Manag. Quart. 16, 148–171. 10.1080/16184742.2016.1140797

[B46] Zhu W. Feng J. Li C. Wang H. Zhong Y. Zhou L.. (2021). COVID-19 risk assessment for the Tokyo Olympic Games. Front. Public Health. 9:730611. 10.3389/fpubh.2021.730611 34760863 PMC8572808

